# GP knowledge, attitudes, beliefs, and practices surrounding the prescription of e-cigarettes for smoking cessation: a protocol for a mixed-method systematic review

**DOI:** 10.3399/BJGPO.2021.0091

**Published:** 2021-11-24

**Authors:** Melis Selamoglu, Bircan Erbas, Karthika Kasiviswanathan, Chris Barton

**Affiliations:** 1 Department of General Practice, School of Public Health and Preventive Medicine, Monash University, Melbourne, Australia; 2 Department of Public Health, School of Psychology and Public Health, La Trobe University, Melbourne, Australia; 3 School of Public Health and Preventive Medicine, Monash University, Melbourne, Australia

**Keywords:** e-cigarettes, electronic nicotine delivery systems, smoking cessation, primary health care, general practice, vaping

## Abstract

**Background:**

Electronic cigarettes (e-cigarettes) are being marketed to people who smoke (PWS) as a smoking cessation aid. GPs have an important role in providing patients with support to encourage them to quit smoking. The emergence and marketing of e-cigarettes as a smoking cessation alternative poses challenges to GPs in advising and supporting PWS to quit.

**Aim:**

This systematic review aims to synthesise available evidence on the knowledge, attitudes, and perceptions of GPs about e-cigarettes as a smoking cessation aid.

**Design & setting:**

Mixed-methods study review including quantitative, qualitative, and mixed-methods studies of GPs in primary care settings.

**Method:**

MEDLINE, CINAHL (Cumulative Index to Nursing and Allied Health Literature), Scopus, PsycINFO, and Embase databases will be searched to identify articles published between 1 January 2003 and 30 June 2021. A Google search will be conducted to identify grey literature. Two independent reviewers will screen abstracts for relevance and full-text studies. Articles will be appraised for quality using the Mixed Methods Appraisal Tool (MMAT) and a Preferred Reporting Items for Systematic Reviews, and Meta-Analysis (PRISMA) diagram will illustrate the flow of articles and reasons for exclusion. An evidence synthesis method will be employed and guided by the Theory of Planned Behaviour (TPB). A descriptive qualitative synthesis of the findings will be reported.

**Conclusion:**

Findings will provide a synthesis of current evidence regarding the knowledge, attitudes, and perceptions among GPs of e-cigarettes as a smoking cessation aid. This information will be useful to guide future research on the needs of GPs in advising and supporting patients to quit smoking. It will also assist in the development of health policy and guidelines on the role and place of e-cigarettes as a smoking cessation aid.

## How this fits in

GPs have an important role in providing patients who smoke with information, support, and treatment to encourage them to quit smoking. There is limited evidence supporting the efficacy of e-cigarettes in smoking cessation and their use could possibly affect future quit attempts. Despite this, in some countries e-cigarettes are endorsed as a harm reduction tool and are recommended as a smoking cessation aid, whereas in others, their use is heavily discouraged. The findings from this systematic review will be useful to guide future research on the needs of GPs in advising and supporting patients to quit smoking. It will also provide evidence for health policy and guidelines on the role and place of e-cigarettes as a smoking cessation aid.

## Introduction

E-cigarettes or electronic nicotine delivery systems (ENDS) are battery-powered products that heat liquids (e-liquid) to produce an aerosol that can be inhaled by the consumer.^1–3^ This is also known as ‘vaping’. They come in two formats, ‘open’, which are refillable, and ‘closed’, which are disposable or refilled using cartridges.^2^ E-cigarettes have been marketed to both younger and older PWS as a safer, cheaper, and healthier option to smoking, and have been promoted as a smoking cessation tool.^4,5^

The World Health Organization (WHO) reported in 2018 that 41 million people globally have used some form of e-cigarette.^1^ This represents a staggering increase from 2011, when it was estimated there were just 7 million users.^1^ Euromonitor International anticipates that this figure will reach over 55 million users by 2021.^6,7^ Although these products do not contain tobacco, they do contain nicotine, propylene glycol, glycerine, flavouring agents, and toxic chemicals that have been shown to be harmful to health,^1,2,8^ and may be linked to conditions such as lung disease, cardiovascular disease, and cancer.^9,10^

Data from the Special Eurobarometer 458,^11^ surveyed 27 901 participants aged >15 years from 28 member states in the European Union (EU) on tobacco consumption, and found that the most popular reason among consumers for starting using e-cigarettes was to aid with quitting smoking or to reduce the amount of tobacco consumed. Similarly, the National Drug Strategy Household Survey 2019, conducted by the Australian Institute of Health and Welfare (AIHW),^12^ reported that among Australian PWS aged >14 years, 32% had tried e-cigarettes to help with quitting smoking. In addition, the general population of PWS surveyed perceived e-cigarettes: to be cheaper, to be less harmful, to taste better than standard cigarettes, to aid with cutting down on the number of cigarettes smoked, and to help PWS from going back to smoking regular cigarettes.^11,12^

The proportion of adults aged >18 years who are currently using ENDS is still comparatively low compared with those smoking cigarettes. Among select English speaking countries, the highest prevalence of current e-cigarette use among adults is in the UK (6.2%),^10,13^ followed by New Zealand (3.8%),^10^ the US (3.2%),^14^ Australia (2.5%),^12^ and Canada (1%).^15^ Comparatively, in the EU, a quarter of all young people had tried e-cigarettes, which was slightly higher than adults aged 25–39 years (21%) and just fewer than half of PWS (47%) had tried to quit smoking using e-cigarettes.^11^

Little is known about GPs' preparedness to have discussions with their patients and their intentions to prescribe e-cigarettes as a smoking cessation tool. Many physicians in the US lacked confidence in their knowledge of e-cigarettes and the skills to effectively communicate with their patients about their use, indicating that more evidence and information is needed around the use of e-cigarettes for smoking cessation.^16^ Moreover, studies from Egnot *et al* *,* ^17^ El-Shahawy *et al,* ^18^ and Stepney *et al*,^19^ reported similar concerns about recommending e-cigarettes as an aid to smoking cessation owing to the modest evidence of efficacy and safety around the product. Despite this, these devices are being marketed by manufacturers as a smoking cessation aid and are promoted as safer alternatives to regular cigarettes.^1^

Differences in community uptake of e-cigarettes are likely influenced by the very different policy and regulatory approach to ENDS in some countries. In the UK, 2 million PWS had used e-cigarettes to stop smoking in 2016 and a further 470 000 PWS had taken up e-cigarettes as a form of smoking cessation.^20^ E-cigarettes are legal in the UK and the government has been supporting PWS to take up new and *‘* *less harmful* *’* nicotine products, such as e-cigarettes, to aid with quitting smoking.^20^ In contrast, healthcare professionals in Australia are not able to prescribe e-cigarettes, but PWS are able to buy non-nicotine e-cigarettes from vape shops and online stores. E-liquid containing nicotine is banned from sale in Australia, but it can be obtained with a valid prescription from a healthcare professional as of 1 October 2021.^8,21^

In the US, one study found that between 2014 and 2016 a quarter of PWS had replaced regular cigarettes with e-cigarettes in their latest quit attempt, yet the Food and Drug Administration has not approved e-cigarettes as a safe alternative to smoking.^22^ The US Preventive Services Task Force^23^ and the WHO^1,10^ state that, due to insufficient evidence around e-cigarettes and hesitation around e-liquid products, they are not recommended for adults as a means for smoking cessation, and it is recommended that physicians inform their patients of the possible harms of e-cigarettes.

This review is being conducted at a time when laws and clinical practice guidelines regarding the use of e-cigarettes are struggling to keep up with changing societal attitudes towards ENDS and the increasing use of e-cigarettes in the community. This is occurring in the context of promotion and marketing of these devices as a smoking cessation aid. It is therefore timely to synthesise current literature describing the knowledge, attitudes, and perceived efficacy of GPs discussing e-cigarettes with their patients to support smoking cessation. This information will be useful for the development of guidelines internationally and nationally on the potential role of e-cigarettes as a smoking cessation aid, and the needs of GPs in supporting patients to make decisions about the use of e-cigarettes.

## Method

This review will be conducted and reported in compliance with the PRISMA guidelines.^24^

The TPB^25^ will be used as a framework to explore and understand prescribing intentions and behaviours of healthcare professionals. The TPB is based on the premise that individuals make logical, reasoned decisions to engage in specific behaviours by evaluation of the information available to them.^26^ It comprises of three domains that are used to predict intentions, which in turn are determinants of behaviour. The domains include: (i) attitudes and beliefs; (ii) subjective norm and the influence of social pressure; and (iii) perceived behavioural control.^25,27,28^ The TPB as a framework has been used extensively to understand and model smoking and smoking cessation,^29^ and can help understand GPs intentions to prescribe treatments such as e-cigarettes as a smoking cessation aid. There has only been one review to date by Erku *et al,* ^30^ which investigated the beliefs of healthcare professionals regarding ENDS. However, this systematic review grouped all health professionals including allied health providers and didn’t incorporate theory, making it difficult to translate the findings to primary care.

### Search strategy

The search will be conducted in English. Peer reviewed articles published between 1 January 2003 and 30 June 2021, with no limit to countries, will be considered. A subject librarian at Monash University was consulted to assist the authorship team in constructing the search strategy and to identify relevant databases to search. The final set of search terms used for the search of OVID MEDLINE are provided in Supplementary Table S1. Searches of the other databases will be adapted from this search.

The following databases will be searched for peer reviewed articles that meet the eligibility criteria:

- MEDLINE
- CINAHL
- Embase
- Scopus
- PsycINFO

In addition, Google will be searched to identify any additional peer reviewed literature, as well as grey literature. Reports from peak bodies, government, and non-government organisations that appear on the first 10 pages of the search results will be accessed. Grey literature is an important source as it may include relevant reports from policymakers, researchers, and healthcare professionals addressing issues of policy, procedure, and guidelines.^31^

Additionally, citations of included studies will be searched and hand-searching of reference lists will be conducted to identify any further articles not identified in the electronic search.

### Study selection

Covidence will be used to support the review and selection of articles for data extraction. Results from the searches will be uploaded and duplicates removed. The abstract and titles of all articles will be assessed for relevance and any that clearly do not meet the inclusion criteria will be removed. Two reviewers (MS and KK) will independently conduct the title and abstract screening. Any conflicts will be resolved by discussion between the reviewers. If needed, a third author (CB) will be consulted. The primary author will then download and review the full text of these articles and assess them to determine if they meet the inclusion criteria.

#### Inclusion criteria

Peer reviewed articles that have been published in journals included in the databases listed above, and other studies that are identified will be included. The studies will need to collect quantitative, qualitative, or mixed-methods data to determine knowledge, attitudes, social norms, and perceived behavioural control of GPs (primary care doctors or family physicians or their equivalent) for use of e-cigarettes, or vaping, as a harm reduction tool or for smoking cessation. Studies with a primary focus on these issues for GPs of any age, country, and level of experience will be included in the review.

#### Exclusion criteria

Articles that are in a language other than English, reviews or editorials, letters, commentary and opinion or perspective pieces will be excluded from the study. Conference proceedings and abstracts without full text will be excluded.

The selection of studies for inclusion will be reported using a PRISMA flow diagram^24^ to illustrate the study selection process.

### Quality appraisal

Appraisal of studies will be undertaken using the MMAT. MS will assess the quality of studies as high, medium, or low using published MMAT criteria.^32,33^ Articles will not be excluded based on their rating, but the rating will be used to guide the degree to which outcomes of individual studies influence the overall interpretation of findings.

### Data extraction and synthesis

Data will be extracted using a customised data extraction form (Supplementary Table S2). Data will be extracted by the lead author (MS). A second author (CB) will independently extract data from the first 20% of articles to check accuracy. The authors will review this and discuss discrepancies before extraction of data from the remaining articles. Data will include study demographics, author(s), publication year, place of study, study design, context and setting, response rate, and outcome or intervention measures.

An evidence synthesis method^34^ will be used to combine evidence from different studies. Outcomes from qualitative and quantitative studies will be grouped according to domains within the TPB (knowledge and attitudes, social norms, perceived behavioural control, and prescribing intentions or practices). A meta-analysis of quantitative studies will not be conducted as most studies differ in terms of methodological approach, study aims and objectives, and survey items. The extracted data will be grouped into sub-categories of the TBP and reported narratively. Variation between countries with different policy settings will be explored. Similarly, findings from qualitative studies will be grouped as above and relevant findings synthesised and reported descriptively. Findings from qualitative and quantitative studies will be given equal weighting in the interpretation of data.

## Discussion

This systematic review will provide a synthesis of evidence from published peer reviewed studies and grey literature about knowledge, attitudes, and perceptions of GPs towards e-cigarettes as a smoking cessation aid. This will provide information and guidance to support policymakers, healthcare professionals, and tobacco-control researchers about GPs' needs and recommendations to support patients when discussing e-cigarettes and their use for smoking cessation.

Despite modest evidence on the effectiveness of e-cigarettes as a smoking cessation tool,^35,36^ many GPs internationally appear optimistic about e-cigarettes as an alternative to smoking tobacco, believing that e-cigarettes can promote smoking cessation.^35^ Though these products may have lower risks compared with combustible cigarettes, they are not entirely risk free.^1^

It is important for GPs to understand the safety and efficacy of e-cigarettes so that effective recommendations can be given to patients if they do wish to use e-cigarettes, or if they are to be recommended or prescribed as a smoking cessation treatment. GPs are unsure around the efficacy and safety of e-cigarettes;^17,18,37–40^ however, some findings have revealed that GPs believe e-cigarettes are safer than combustible cigarettes.^41,42^

Current available literature has revealed that GPs seek information and guidance from online government sources and reported that patients were more informed about e-cigarettes than their GPs.^19,37^ This suggests that there is knowledge deficit around e-cigarettes for smoking cessation, and further research and guidance is needed to provide patients with correct information regarding e-cigarettes as a smoking cessation aid in primary care settings.^16–19,37,38,41,43–46^

This systematic review will synthesise evidence from published, peer reviewed studies and grey literature, to guide policymakers, healthcare professionals and tobacco-control researchers about the knowledge, attitudes, and practices of GPs with respect to e-cigarettes and their role and place as a smoking cessation aid.
